# Aversion to Hospital Admission Due to Fear of COVID Infection Leading to Fatality From Diabetic Ketoacidosis

**DOI:** 10.7759/cureus.20251

**Published:** 2021-12-07

**Authors:** Hasina Mohammad Ashraf, Trisha Sunderajan, Louisdon Pierre, Noah Kondamudi, Adebayo Adeyinka

**Affiliations:** 1 Pediatrics, Children's Hospital Of Michigan, Detroit, USA; 2 Pediatrics, The Brooklyn Hospital Center, Brooklyn, USA

**Keywords:** future educational reform, family education, hospital aversion, covid 19, diabetic ketoacidosis (dka)

## Abstract

Diabetic ketoacidosis (DKA) is a potentially life-threatening condition that occurs in patients with diabetes mellitus (DM) where the decrease in the insulin level leads to a state of metabolic acidosis and hyperglycemia. Based on the literature review, the risk of severity of DKA in children was significantly associated with coronavirus disease-2019 (COVID-19) cases during the first wave of the pandemic. This could be attributed to social distancing restrictions which delayed hospital presentation and timely treatment and interventions. We present the case of a 15-year-old female, with non-insulin-dependent diabetes (type 2), who presented during the COVID-19 pandemic with severe DKA from another hospital. She had elevated glucose level at home for three days that was worsening but her parents continue to manage the patient at home out of fear of the patient contracting COVID-19 if she was brought to the hospital. After she deteriorated, the parents took her to the nearest hospital which did not have a pediatric intensive care unit (PICU). She was immediately transferred to our facility. The patient was intubated immediately on arrival because of altered mental status possibly due to cerebral edema from severe metabolic acidosis and elevated glucose level. The patient rapidly progressed into shock, acute respiratory distress syndrome (ARDS), and multiple organ dysfunction syndrome (MODS). She was managed aggressively with vasopressors, fluid resuscitation, and insulin drip. She had four cardiac arrests for which she was resuscitated. Despite all efforts, she subsequently expired less than 24 hours after admission. We intend on shedding light on an emerging phenomenon due to the ongoing COVID-19 pandemic, wherein due to the fear of contracting COVID-19, many parents opt to keep and manage sick children at home. This report highlights the important role that the aversion of presenting to medical establishments out of fear of contracting COVID-19 may have led to the untimely and preventable death of our patient. It also outlines the importance of future educational reforms toward changing the patient and family’s perception of hospitals and medical institutions, especially in children with pre-existing chronic medical conditions.

## Introduction

In December 2019, news broke of a rapidly spreading novel virus, severe acute respiratory syndrome coronavirus 2 (SARS-CoV-2), that was causing a deadly pneumonia. With the number of cases and the countries affected rising exponentially, travel bans and stay-at-home lockdown orders were issued by many governments around the world [1,2]. New information about complications and the rising death toll had been on a constant stream through most media outlets throughout the country [3] While telling citizens to stay at home to minimize contact is touted as the way to “flatten the curve” in the pandemic, there have been some unexpected consequences despite the stay-at-home order [4].

Diabetic ketoacidosis (DKA) is a state of severe insulin deficiency leading to acidosis that can develop most commonly in patients with type 1 diabetes mellitus (DM) [5,6] but also seen, less frequently, in type 2 DM, i.e about 10%-30% [7].

Several fatal complications, like cerebral edema, pulmonary edema, acute respiratory distress syndrome (ARDS), electrolyte imbalances, venous thrombosis, and acute pancreatitis can occur with DKA [8]. ARDS and respiratory failure are rare complication of DKA. Conditions that impair respiratory function triggered by DKA can be detected at onset but are usually more common during treatment. These include deficits of potassium, phosphate, magnesium and hydrostatic or non-hydrostatic pulmonary edema [8,9].

Diabetic patients and families of young diabetics receive diabetic education from their primary doctors and endocrinologists about associated health risks. While families may be aware of these dangers, the urgency of early intervention, especially in the case of development of DKA, is imperative for a good outcome. 

## Case presentation

A 15-year-old female, known type 2 diabetic patient was emergently transferred to our pediatric intensive care unit (PICU) from the emergency department of a local hospital for management of DKA with worsening mentation. The patient was diagnosed with type 2 diabetes at the age of ten years . Her diabetes was primarily managed by a pediatric endocrinologist, who had started her on daily metformin. Upon further inquiry with the grandmother, the patient was non-compliant with her medications, often hiding her pills away. She had also been lost to follow up and her last visit with the endocrinologist was in 2018. The patient had been admitted to the hospital several times in the past due to hyperglycemia without DKA but never to the PICU, and never requiring respiratory support. For three days prior to this current admission, the mother endorsed that the patient was not feeling well. The patient’s mother was convinced that the patient was just exhausted because she had been staying up late to play video games every night since the quarantine. 

While at home, on the day of admission, the patient was noted to be breathing abnormally and was lethargic. When the family had attempted to wake her up in the morning, she refused to get out of bed and would repeat that she was hungry. At that point, the mother became concerned and called emergency medical services (EMS). On arrival at the emergency room, the initial blood glucose was found to be 401mg/dL and a normal saline (NS) bolus was given. Laboratory results indicated she had a severe anion gap metabolic acidosis with arterial blood gas (ABG) showing pH <6.8, pCO2 21mmHg, pO2 50mmHg, HCO3 unreadable. The basic metabolic panel was significant for hyponatremia with sodium of 134meq/L, and bicarbonate of 3meq/L, leading to an anion gap of 28. She had also developed acute kidney injury (AKI) secondary to severe dehydration and had a creatinine of 11mg/dL. Complete blood count (CBC) was remarkable for leukocytosis (24.7) and monocytosis (14.7). After the NS bolus, she was started on NS 0.9% infusion at 150ml/hr (one and half maintenance) and regular Insulin infusion at 0.1 units/ kg before being transferred to our PICU.

On arrival at the PICU, the patient was found to be unresponsive with a Glasgow Coma Scale of 3. In order to protect the airway, she was intubated within 30 minutes of arrival. She was on ventilator support mode of synchronized intermittent mandatory ventilation-pressure regulated volume control (SIMV-PRVC). Based on her follow-up examination and the repeated arterial blood, the patient was found to be in severe DKA with possible underlying cerebral edema. She was started on a hydration regimen of NS and an insulin drip.

Approximately four hours after she had been stabilized, her condition deteriorated and the patient progressed from pulseless ventricular tachycardia to ventricular fibrillation. During the code, she was given four doses of epinephrine, four doses of sodium bicarbonate, one dose of amiodarone, two doses of calcium chloride, and the patient was defibrillated four times after which there was return of spontaneous circulation (ROSC). ABG during the code showed pH was 6.8 and HCO3 was below 5, blood glucose was 419. EKG was performed and showed sinus tachycardia and prolonged QTc of 470ms. An hour after the initial code, the patient became bradycardic, then went into pulseless electrical activity (PEA). High-quality cardiopulmonary resuscitation (CPR) was initiated and after receiving two doses of sodium bicarbonate and one dose of epinephrine, there was ROSC. The patient was started on a sodium bicarbonate drip to correct severe metabolic acidosis. Due to her rapid deterioration, there was a concern about possible underlying coronavirus disease-2019 (COVID-19). A SARS-CoV-2 RNA polymerase chain reaction (PCR) was sent twice, both times from respiratory secretions from the endotracheal tube. Both tests were negative. The trend of her blood gas is shown in Table 1 and the trend of her electrolytes is shown in Table 2.

**Table 1:** Blood gases tracked over the course of the admission.

pH	6.8	6.89	6.85	6.95	
pCO2 mmHg	40	65	53	60	
pO2 mmHg	<36	30	32	91	

**Table 2:** Complete metabolic panel/basic metabolic panel over the course of the illness. BUN: blood urea nitrogen; AST: aspartate aminotransferase; ALT: alanine aminotransferase; ALP: alkaline phosphatase.

Complete and Basic Metabolic Panel							
Sodium mEq/L	134	131	140	137	138	131	142
Potassium mEq/L	5.2	5.5	3.4	3.5	3.4	3.8	3.2
Chloride mEq/L	103	113	115	113	115	114	118
Bicarbonate mEq/L	Undetectable	5	8	10	12	11	13
BUN mg/dl	11	20	22	24	25	28	34
Creatinine mg/dl	1.19	1.8	2	2.2	2.1	2.4	3.5
Glucose mg/dl	465	436	442	561	519	527	502
Calcium mg/dL	9.4	8	10.4	8.3	7.1	7.2	6.8
Anion Gap	28			14			
AST U/L	30	28				>717	>717
ALT U/L	12	11				175	137
ALP U/L	298	212				131	107
Albumin g/dL	4	3.3				2	1.8
Phosphorous mmol/L		3.8	4.3	4.3	3.5	3.4	1
Magnesium mmol/L		1.8	1.8	1.5	1.3	1.2	1.5

The patient had two more cardiac arrests and subsequently expired despite aggressive resuscitative efforts.

## Discussion

The development of DKA in patients with both type 1 and, less frequently, type 2 diabetes is a well-known and studied phenomenon [10]. The treatment for DKA relies on the principle of rehydration, resolving the hyperglycemic state, correcting any electrolyte imbalances, and transitioning out of a state of metabolic acidosis [11,12]. Timely management of DKA to prevent complications like cerebral edema, ARDS, shock, AKI, and venous thrombosis is key to preventing morbidity and mortality. Our patient presented to the emergency room about 72 hours after the onset of symptoms because of the parental fear of the patient contracting COVID-19 infection. The severe metabolic acidosis and electrolyte imbalance caused a rapid progression to ARDS and respiratory failure. The altered mental status and a Glasgow Coma Score of 3 on admission to our PICU highly suggest that she also developed cerebral edema as a complication although we did not get a CT scan of the brain due to her hemodynamic instability. Cerebral edema is the most dreaded complication associated with DKA. The risk of deteriorating and developing cerebral edema is 0.5%-1%. While the development of cerebral edema is a relatively rare complication, there is high mortality with one estimate showing up to 40%-90% [13,14].

Cerebral edema is associated with about 50% to 60% of deaths related to diabetes in children [15].

According to the Centers for Disease Control (CDC), as of April 20, there have been 746,625 cases of COVID-19 with 39,083 dead from confirmed and probable cases. With the number of cases and deaths rising, the governor of the state of New York issued a statewide “pause” to be effective on March 22. There were guidelines set to decrease gatherings, social interactions and for those who were ill to stay at home and contact their doctor for a tele visit before going to a clinic or hospital. With this order, there had been a dramatic decrease in patient visits to the pediatric emergency department and clinics. The question arises about what is happening with the patients who regularly show up in our emergency department or urgent care. With hospital visits there is always an inherent risk of exposure to someone with a communicable disease; the COVID-19 pandemic has made this possibility become a reality for many people. There may have also been a role to play with the stigmatization of seeking medical care when the illness is perceived as minor. However, in the case of patients with chronic medical conditions, minor illnesses can trigger fatal sequelae. 

The field of pediatrics is differentiated from the other specialties in that pediatric patients are not the primary decision-makers [16]. The choice of whether to go to the hospital when they are feeling ill is one that is made by the parent or guardian. In the case of our patient, even though she was not feeling well, the difficult decision had to be made of whether to care for her at home or if her illness warranted taking her to an environment where there may be exposure to the SARS-CoV-2 virus. This decision was further confounded by the fact that she was an adolescent who was already non-compliant with her medications, so whether she took her illness seriously is definitely in question.

## Conclusions

We postulate that fear may have been a significant underlying factor in determining when the patient presented to seek medical attention. The lesson to be taken away for future outbreaks is the necessity of development of strategies that will balance public health protection while dispelling fear among patients who need to seek medical attention. As the dust settles, the collateral damage from placing the world on quarantine will come to the fore, and more cases like that of our patient will be elucidated.
